# Environmental Influences on Food Addiction and Cardiometabolic Profiles in Law Enforcement Officers

**DOI:** 10.3390/ijerph23030311

**Published:** 2026-03-01

**Authors:** Yunzhi Qian, Grace E. Russell, Ziyuan Shi, Ya-Ke Wu

**Affiliations:** 1 Department of Nutrition, University of North Carolina at Chapel Hill, Chapel Hill, NC 27599, USA; 2 School of Nursing, University of North Carolina at Chapel Hill, Chapel Hill, NC 27599, USA; 3 Department of Psychiatry, University of North Carolina at Chapel Hill, Chapel Hill, NC 27599, USA

**Keywords:** law enforcement, environmental factors, food addiction, cardiometabolic profiles

## Abstract

**Highlights:**

**Public health relevance—How does this work relate to a public health issue?**

- This study addresses how adverse local food environments may exacerbate food addiction and cardiometabolic risk among law enforcement officers, a high-stress occupational group with elevated vulnerability to cardiovascular disease.

**Public health significance—Why is this work of significance to public health?**

- Findings demonstrate that poorer county-level food environments, particularly in rural areas, are associated with greater food addiction symptoms; these are, in turn, associated with unfavorable cardiometabolic profiles, highlighting potential environmental influences on cardiovascular disease risk beyond individual behaviors.

**Public health implications—What are the key implications or messages for practitioners, policy makers and/or researchers in public health?**

- Although preliminary and non-causal, our findings underscore the complex role of environmental influences on disordered eating in law enforcement and highlight the need for more fine-grained, individual-level assessments of environmental exposure and prospective, longitudinal research to inform effective, context-specific interventions.

**Abstract:**

Law enforcement officers experience substantial occupational stressors that increase vulnerability to food addiction and cardiovascular disease (CVD), which may be compounded by adverse local environments. This study examined associations among county-level environmental factors, food addiction, and cardiometabolic profiles among North Carolina law enforcement officers. Participants included 330 officers (mean age = 37.98 years; mean BMI = 30.53 kg/m^2^) who completed the Yale Food Addiction Scale 2.0 and underwent assessments of anthropometrics, blood pressure, blood lipids, and glucose. County-level Food Environment Index (FEI) scores and counts of fast-food restaurants, recreation and fitness facilities, and crime events were obtained from public data sources. Comparative analyses evaluated differences by county type and region, and BMI- and sex-adjusted regression models assessed associations among environmental factors, food addiction symptoms, and cardiometabolic profiles. Rural counties had significantly poorer FEI scores than suburban and urban counties, and rural officers reported the highest food addiction symptoms. Lower FEI scores were significantly associated with greater food addiction symptoms, which were, in turn, associated with higher adiposity and lower triglyceride levels. The findings support associations between food addiction and CVD risk, while underscoring potential influences of food environments on food addiction, warranting further investigation using more precise and up-to-date measures.

## 1. Introduction

Law enforcement is a unique demanding profession with intense levels of operating and organizational stress, increasing officers’ risk for cardiovascular disease (CVD) over that of the general population [1,2,3,4]. This disparity places officers at a higher lifetime risk for hypertension, obesity, metabolic syndrome, and dyslipidemia, which contributes to increased risk of CVD [5,6,7]. The prevalence of these conditions is alarmingly high among law enforcement; for example, large-scale studies have found a metabolic syndrome prevalence of over 23% in police cohorts and a central obesity prevalence of 50%, with component risk factors like elevated triglycerides affecting nearly 41% of officers [6,8,9]. Further, these risks are not evenly distributed across the profession, with differences in risk profiles reported between men and women. Male officers typically present with a more adverse cardiometabolic profile, including a higher prevalence of metabolic syndrome, significantly higher blood pressure, and greater dyslipidemia compared to female officers [5,10,11]. By contrast, female officers have demonstrated a higher risk for cardiac arrhythmia (Standardized Incidence Ratio of 2.60 for female vs. 1.73 for males) [12]. These sex-specific health trajectories are often underpinned by different maladaptive coping strategies. For instance, male and female officers exhibit distinct patterns of disordered eating in response to occupational stress, with female officers reporting significantly higher levels of restricting and purging, while male officers report higher levels of binge eating and muscle-building behaviors [11]. These stress-induced behaviors can escalate into more severe conditions such as food addiction, a compulsive pattern of overeating that directly promotes obesity and insulin resistance, thereby accelerating the premature development of CVD [13,14].

Food addiction is characterized by compulsive overconsumption of highly palatable, energy-dense foods (i.e., those rich in sugars, fats, and refined carbohydrates), which can lead to excessive caloric intake and poor dietary quality and stimulate reward pathways in the brain similarly to addictive substances [15]. A meta-analysis found that approximately 24% of adults met criteria for food addiction by the Yale Food Addiction Scale (YFAS) 1.0 and 2.0, with even higher rates (55%) among individuals with binge-eating disorder [16]. Similar to substance use disorders, individuals with food addiction exhibit patterns of craving, loss of control, and continued use despite adverse consequences [17], resulting in significant physical health complications such as obesity [18], type 2 diabetes [19], elevated blood pressure [20], and dyslipidemia [21], as well as psychological distress including guilt, shame, depression, and anxiety [22,23].

Importantly, the modern food environment—saturated with inexpensive, ultra-processed foods and aggressive food marketing—amplifies exposure to such addictive foods [24]. Individuals living in low-income neighborhoods often face limited access to fresh produce and whole foods, while being disproportionately targeted by marketing of fast food and sugary beverages [25,26]. Studies using census tract data demonstrate that wealthier communities tend to have greater physical access to healthier food outlets, whereas economically disadvantaged neighborhoods are often food deserts (i.e., limited access to affordable and nutritious food, particularly fresh fruits and vegetables) or swamps (i.e., unhealthy food options vastly outnumber healthy ones), increasing reliance on processed foods [27]. Concurrently, built environment features such as high crime rates and lack of green space discourage physical activity, further compound CVD risk through sedentary behaviors [28]. While the association between environmental disadvantage and CVD risk has been well established in the general population [29], how environmental factors contribute to food addiction among police officers and how this, in turn, impacts cardiovascular risk remains largely unexplored. Police officers face unique occupational stressors such as shift work, sleep disruption, and chronic exposure to trauma, which may increase vulnerability to disordered eating [30,31]. At the same time, they often work in environments where unhealthy food is readily available and healthier options are limited, such as fast-food outlets during night shifts or vending machines in stations [32]. These combined occupational and environmental pressures may contribute to the development of addictive-like eating behaviors. Thus, understanding how food addiction links environmental exposures to cardiovascular risk in police officers represents a novel and critical research direction, as addressing this overlooked pathway may be essential to reducing cardiovascular health disparities among first responders.

North Carolina (NC), situated in the “Stroke Belt,” a region in the southeastern United States (U.S.) known for its significantly higher rates of stroke and CVD, ranks 6th for stroke mortality and 27th for CVD-related deaths [33,34]. NC’s counties span diverse geographic regions—mountains, piedmont, and coastal—each with distinct cultural, socioeconomic, and infrastructural characteristics that contribute to vast differences in accessing to healthy food resources, availability of recreation and fitness facilities, and crime events. The state is characterized by significant rural-urban heterogeneity, with multiple major metropolitan areas as well as the second-largest rural population in the country [35]. Regional and county-level differences may contribute to varying levels of access to health-promoting resources and prevalence of obesogenic environments, ultimately influencing CVD risk. High quality food environments, for example, are known to be associated with higher diet quality and lower CVD mortality for residents [29,36,37]. In contrast, high density of fast-food restaurants is associated with unhealthy dietary patterns (e.g., high sodium, saturated fats), and proximity to fast food restaurants, both in rural and urban areas, is associated with elevated risk of heart failure [38,39,40]. Additionally, availability of recreation and fitness centers supports physical activity, a protective factor against CVD [41,42]. One recent study found that higher density of fitness and physical activity facilities within a five kilometer radius is associated with a 12% decreased risk of incident CVD [43]. However, such facilities are unequally distributed, with limited availability of physical activity facilities in rural and low-income areas [44,45]. Furthermore, crime levels and workload for police officers also vary across the state. Rural officers carry a disproportionate work burden in their jobs; rural counties average 1.81 law enforcement officers per 1000 NC residents compared to 2.26 in urban areas [46], and the top 50 counties with the highest crime rates are mostly rural [47]. Local and regional differences in crime levels and workload may contribute to chronic occupational stress [48], which is a known CVD risk factor among police officers [9]. However, it is unclear how county-level factors such as access to healthy foods, availability of recreation and fitness facilities, and exposure to crime are associated with individual health outcomes, including food addiction symptoms and cardiovascular risk, among police officers across different county types and regions in NC.

Hence, the purpose of this study was to examine how environmental factors contribute to food addiction and how this, in turn, impacts cardiovascular risk among police officers. The primary aims of the study were as follows: (1) We aimed to assess environmental factors (i.e., county-level Food Environment Index, count of fast-food restaurants, count of recreation and fitness facilities, and count of crime events), food addition symptoms, and CVD-related biomarkers (i.e., body mass index [BMI], body weight, waist circumference, hip circumference, waist-to-hip ratio, systolic blood pressure, diastolic blood pressure, mean arterial pressure, total cholesterol, triglycerides, high-density lipoprotein cholesterol [HDL], low-density lipoprotein cholesterol [LDL], and glucose levels) among NC police officers, considering county types (i.e., urban, suburban, and rural) and regions (Mountain, Piedmont, and Coastal Plain). (2) We aimed to explore how environmental factors associated with food addiction symptoms and how these symptoms, in turn, relate to CVD-related biomarkers. It was hypothesized that lower access to healthy food resources, recreation, and fitness facilities, greater exposure to fast-food restaurants and higher crime events will be associated with increased food addiction symptoms, which will, in turn, be associated with adverse CVD-related biomarkers. The secondary aim explored the potential moderating role of food addiction symptoms in the associations between environmental factors and CVD-related biomarkers among NC police officers. It was hypothesized that the impact of environmental risk factors on CVD-related biomarkers would be more pronounced among individuals exhibiting increased food addiction symptoms.

## 2. Materials and Methods

### 2.1. Study Design and Procedure

Data for this study were drawn from the Carolina Blue Project, a cross-sectional study investigating disordered eating and cardiovascular health in the NC police force. Detailed procedures have been described in a previous study [49]. Participants were recruited through multiple channels, including the project’s official website, targeted social media advertisements, and distributed flyers. Those who met the eligibility criteria provided informed consent electronically and completed 14 questionnaires online survey using the REDCap platform. Following survey completion, participants had the option to attend an in-person health assessment, which included evaluations of cardiovascular risk factors such as height, weight, blood pressure, and fasting lipid and glucose levels. Upon fulfilling all study requirements, participants were compensated with a USD 100 gift card. Data were collected between 1 February 2023, and 21 February 2025. The study protocol received ethical approval from the University of North Carolina at Chapel Hill Institutional Review Board (Approval No. 22-2052).

### 2.2. Participants

Participants were eligible for inclusion if they were current law enforcement officers in NC, aged 18 years or older. As the collection of blood samples poses an increased risk of bleeding, officers with known coagulation disorders were excluded from the study.

### 2.3. Measurements

#### 2.3.1. Demographics

Participants self-reported demographic information including age, biological sex, race, ethnicity, and work county. The participants’ self-reported work county was classified as urban (i.e., an average population density exceeding 750 people per square mile), suburban (i.e., an average population density between 250 and 750 people per square mile), or rural (i.e., an average population density of 250 people per square mile or less) [50]. There are 100 counties in NC (6 urban, 16 suburban, and 78 rural counties). We also grouped the counties into three regions by geographic location: Mountains, Piedmont, and Coastal Plain [50]. We did not collect data on actual patrol routes or officers’ place of residence due to safety and confidentiality concerns. Disclosing residential locations or specific patrol routes could place officers at risk of retaliation from individuals involved in criminal activity and could compromise both officer safety and operational security. Limiting geographic data to the work county allowed us to capture relevant environmental exposures while protecting participants.

#### 2.3.2. County-Level Environmental Factors

The Food Environment Index data were sourced from the National Institute on Minority Health and Health Disparities website and reported at county level [51]. This index, ranging from 0 (poorest conditions) to 10 (most favorable), equally incorporates two key dimensions of the food environment at the county level from 2019 to 2022: limited access to healthy food options and food insecurity. The measure of limited access is made available by the U.S. Department of Agriculture Economic Research Service’s Food Environment Atlas [52], and reflects the proportion of individuals who are both low-income, defined as earning no more than 200% of the federal poverty level for their household size, and who reside at a considerable distance from a grocery store. For urban (nonrural) areas, this threshold is more than one mile, while for rural areas, it is more than ten miles. Food store locations are identified using merged directories of SNAP-authorized supermarkets and large grocery stores meeting minimum sales and department criteria and geocoded for spatial analysis [52]. Population and income data are drawn from the U.S. Census and American Community Survey. Distances to the nearest food store are calculated at the one-half kilometer square level. Rural or urban status is designated by the Census Bureau’s definition. Food insecurity, the second component of the Food Environment Index, captures the share of the population that lacked consistent access to adequate food over the past year [53]. County-level food insecurity rates incorporated into the Food Environment Index are estimated by Map the Meal Gap from Feeding America using a two-stage fixed effects model, drawing on data from the American Community Survey, the Community Population Survey, and the Department of Labor Statistics [52].

Data on the number of fast-food restaurants as well as recreation and fitness facilities per NC county from the most recent year available, 2016, were sourced from the U.S. Department of Agriculture Economic Research Service’s Food Environment Atlas website [52]. The Atlas compiles over 300 indicators of the food environment from multiple sources and organizes them into three broad categories: food choices, health and well-being, and community characteristics. In the current study, we use the 2016 county-level counts of fast-food restaurants and recreation and fitness facilities across NC in the analysis. Both restaurant data and recreation and fitness center data come from the U.S. Department of Commerce, Bureau of the Census, County Business Patterns [52]. Fast-food restaurants, classified under North American Industry Classification System code 722513, are establishments that primarily provide limited-service food, where customers pay before eating and food is consumed on site, taken out, or delivered [52]. Fitness and recreation centers, defined by North American Industry Classification System code 713940, include establishments focused on exercise and other physical conditioning or sports activities, including swimming, skating, or racquet sports [52].

The number of reported crime events, including murder, rape, robbery, aggravated assault, burglary, larceny, and motor vehicle theft, for each NC county in the most recent year available, 2023, was obtained from the NC State Bureau of Investigation. These data reflect incidents reported by local law enforcement agencies across the state [54].

#### 2.3.3. Food Addiction Symptoms

Food addiction symptoms were measured using a questionnaire in the online REDCap system. The questionnaire was designed using Yale Food Addiction Scale Version 2.0 (YFAS 2.0) [55]. The YFAS 2.0 is a 13-item self-report questionnaire with Likert-type responses (0 = never, 1 = less than monthly, 2 = once a month, 3 = 2 to 3 times a month, 4 = once a week, 5 = 2 to 3 time a week, 6 = 4 to 6 times a week, 7 = every day) designed to assess addiction-like eating behaviors related to highly processed, high-calorie foods [55]. It aligns with the Diagnostic and Statistical Manual of Mental Disorders, Fifth Edition (DSM-5) criteria for substance use disorder, including the addition of craving and the merger of abuse/dependence criteria, making it a modern and clinically relevant tool to assess food addiction symptoms. Each item was converted into threshold variables. For example, if the score for item one is greater than or equal to 4, then the threshold is set at 1, indicating that the criterion has been met. After calculating these threshold variables, all items, except for clinical items 5 and 6, were summed to create a total count of food addiction symptoms, which can range from 0 to 11 symptoms. The YFAS 2.0 has shown Kuder–Richardson alpha of 0.90 [55]. The convergent validity (r) between the YFAS 2.0 and other problematic eating behavior measures have ranged from 0.24 to 0.63 (all *p* < 0.01) [55].

#### 2.3.4. CVD-Related Biomarkers

The participants’ height and weight, BMI, waist and hip circumference, waist-to-hip ratio, and blood pressure were measured by a trained research assistant. All participants were asked to wear light clothing for the measurement, which used a portable Martin stadiometer. Each measurement was taken twice and averaged. BMI was calculated using the standard formula (i.e., BMI = weight (kg)/height (m)^2^). Waist and hip circumference were measured using standard tape techniques, and the waist-to hip ratio was computed as waist circumference divided by hip circumference. After a 5 min rest, seated blood pressure was measured according to the American Heart Association guidelines [56]. Mean arterial pressure (MAP) was calculated using the standard formula:

MAP = [systolic blood pressure + (2 × diastolic blood pressure)] ÷ 3.

Fasting (8 h) blood total cholesterol, triglycerides, HDL cholesterol, LDL cholesterol, and glucose levels were measured using the Cholestech LDX™ Analyzer and the Lipid Profile•GLU cassette with 40 μL blood sample via a fingerstick by the trained research assistant [57]. The Cholestech LDX Analyzer measures the levels of total cholesterol (range of values tested: 100–500 mg/dL) and HDL cholesterol (range of values tested: 0–100 mg/dL) by an enzymatic method based on the method formulation of Allain [58] and Roeschlau [59]. The level of triglycerides (range of values tested: 40–650 mg/dL) was measured through an enzymatic method based on the hydrolysis of triglycerides by lipase to glycerol and free fatty acids [60]. The level of glucose (range of values tested: 25–575 mg/dL) was measured using an enzymatic method that employed glucose oxidase to catalyze the oxidation of glucose to gluconolactone and hydrogen peroxide [57]. The Cholestech LDX Analyzer calculated estimated LDL cholesterol (mg/dL) using the Friedewald equation: LDL = total cholesterol − HDL − [triglycerides ÷ 5] [61]. Once the analysis was completed, the cassette and the 40 μL blood sample inside the cassette were discarded. The Cholestech LDX™ System demonstrated good accuracy and precision comparing to venous heparinized whole blood, plasma, and serum samples in a controlled laboratory conditions [62]. The system was calibrated and quality-checked in accordance with the manufacturer’s guidelines [57].

### 2.4. Statistical Analysis

All statistical analyses were conducted using R Statistical Software (v 4.3.0) [63]. Mean and standard deviation were calculated for continuous variables, while frequency and percentage were calculated for categorical variables. Independent samples *t*-tests and one-way ANOVA were conducted to compare numeric variables, including environmental factors and CVD-related biomarkers, across different county types and regions. Pearson’s correlation analysis was conducted to examine the bivariate relationships between pairs of numeric variables. Two-sided *p*-values of less than 0.05 were considered statistically significant.

Prior to conducting the analyses, we undertook a comprehensive assessment of model assumptions and data integrity. The distributional properties of all continuous variables were examined using histograms and descriptive statistics to evaluate adherence to normality. In addition, residual-predicted value plots were inspected for every regression model to verify that the assumption of homoscedasticity was satisfied. These diagnostic checks indicated that the data met the assumptions required for the planned analytical procedures. Across all variables, the proportion of missing data ranged from 0.3% to 52%. Missing values were handled using multiple imputations to preserve statistical power and reduce potential bias [64]. Based on the missingness diagnostics, which included examining patterns of missing data and assessing associations between missingness and observed variables, we determined that the data was consistent with a missing-at-random (MAR) mechanism [65]. Under this assumption, multiple imputations provide more accurate and less biased parameter estimates than methods that analyze only participants with fully observed data [64].

Multiple imputation was performed using multivariate imputation by chained equations (MICE) in R. Ten imputed datasets were generated with 20 iterations per chain using Bayesian linear regression for continuous variables. The imputation model included all variables used in subsequent analyses to preserve associations among variables. Convergence was assessed by monitoring stability of imputed values across iterations. The imputed datasets were combined prior to analysis by averaging continuous variables and applying a majority rule for categorical variables. All imputations were performed prior to conducting the analyses. Given the exploratory nature of this work, we elected not to apply adjustments for multiple comparisons in the analyses. This approach prioritized sensitivity to potential associations while acknowledging that findings should be interpreted with appropriate caution.

Linear regression analyses were conducted to evaluate the associations between environmental factors (independent variables) and food addiction symptoms (dependent variables). Because officers were nested within counties, but many counties had very small sample sizes, all regression models were estimated using county-type clustered robust standard errors based on a heteroskedasticity-consistent (HC1) sandwich estimator to account for within-county-type correlation. Since the YFAS symptom count is a bounded count variable and exhibited overdispersion (variance exceeding the mean), we additionally conducted sensitivity analyses using negative binomial regression to evaluate the robustness of the findings. Both unadjusted models and models adjusted for BMI and sex were estimated. Linear regression coefficient estimates (β), standard error, and confidence intervals (95% CIs) were reported. Environmental factors, including the Food Environment Index, the number of fast-food restaurants, the number of recreation and fitness facilities, and the number of crime events, were examined in separate regression models (one factor per model) to mitigate concerns regarding multicollinearity among these independent variables. BMI was included as a covariate given prior findings indicating a significant association between BMI and the severity of food addiction symptoms [66]. Sex was also included as a covariate, as epidemiologic data indicate that females exhibit a higher prevalence of food addiction compared with males (odds ratio = 3.04, *p* < 0.0001) [67].

The associations between food addiction symptoms (independent variable) and a range of CVD-related biomarkers (dependent variables), including BMI, body weight, waist circumference, hip circumference, waist-to-hip ratio, systolic and diastolic blood pressure, mean arterial pressure, total cholesterol, triglycerides, HDL, LDL, and glucose levels, were evaluated using both unadjusted and adjusted linear regression models. The adjusted models controlled for sex, as it is a well-established determinant of cardiometabolic physiology and has demonstrated significant effects on blood pressure, lipid profiles, and glucose metabolism [68,69].

Moderation analyses were conducted to examine whether the associations between environmental factors (independent variables) and CVD-related biomarkers (dependent variables) varied based on the severity of food addiction symptoms (moderator). Each model included an interaction term between the environmental factor (one for each model), and the food addiction symptom count to assess effect modification. All moderation models, except those where BMI and weight were the dependent variables, were adjusted for BMI and sex to account for potential confounding. For models in which BMI and weight were the dependent variables, only sex was included as the covariate. For each model, we reported the estimated coefficients and corresponding *p*-values for the interaction terms, which indicate whether the strength or direction of the association between the environmental factors and CVD-related biomarkers differs across levels of food addiction symptoms. A significant interaction term was interpreted as evidence that food addiction symptoms meaningfully modify these relationships.

## 3. Results

### 3.1. Participant Characteristics

A total of 330 participants were included in the analysis (Table 1). The mean age was 37.98 years (standard deviation [SD] = 9.06), and two-thirds of participants were male (67.17%). The majority identified as Caucasian (79.08%), followed by African American (14.55%), with small proportions reporting mixed or other racial backgrounds. Most participants were non-Hispanic (93.94%). Approximately 27.27% of participants work in urban counties, 43.33% in suburban counties, and 29.39% in rural counties, with the majority located in the Piedmont Region (63.64%). The mean BMI was 30.53 kg/m^2^ (SD = 6.38), mean waist circumference was 103.06 cm (SD = 16.32), and mean systolic and diastolic blood pressures were 127.15 mmHg (SD = 16.95) and 84.60 mmHg (SD = 11.81), respectively. The mean Food Environment Index score of counties where officers worked was 7.72 (SD = 0.83), and the mean food addiction symptom count was 0.75 (SD = 1.80).

### 3.2. Differences by County Types

Comparisons across county types (Table 2) revealed significant variation in waist circumference (F = 4.67, *p* = 0.03, ŋ^2^ = 0.01) and waist-to-hip ratio (F = 5.38, *p* < 0.01, ŋ^2^ = 0.03), with rural participants having higher waist-to-hip ratio compared to urban and suburban participants. Environmental measures differed significantly across county types. The Food Environment Index was higher in urban and suburban counties than in rural counties (F = 15.89, *p* < 0.01, ŋ^2^ = 0.09). Similarly, urban counties had significantly greater numbers of fast-food restaurants, recreation and fitness facilities, and crime events compared with suburban and rural counties (all *p* < 0.01, ŋ^2^ ranging from 0.57 to 0.72). Food addiction symptoms also differed by county type (F = 4.64, *p* = 0.01, ŋ^2^ = 0.03), with rural participants reporting higher symptom counts compared with urban and suburban participants.

### 3.3. Differences by Region

Analyses across geographic regions (Table 3) showed few differences overall. However, the Food Environment Index varied significantly (F = 5.44, *p* = 0.02, ŋ^2^ = 0.02), with higher Food Environment Index in the Piedmont Region compared to Mountain and Coastal Plain Regions. The Piedmont Region had significantly higher counts of fast-food restaurants, recreation and fitness facilities, and crime events compared to Mountain and Coastal Plain Regions (all *p* < 0.01, ŋ^2^ = 0.12–0.15). Food addiction symptoms were higher among officers in the Mountain Region compared with the Coastal Plain Region (F = 3.50, *p* = 0.02, ŋ^2^ = 0.03).

### 3.4. Correlations Between Environmental Factors, Food Addiction Symptoms, and CVD-Related Biomarkers

Correlation analyses (Table 4) indicated that the Food Environment Index was inversely correlated with food addiction symptoms (r = –0.12, *p* < 0.05). Counts of fast-food restaurants, recreation and fitness facilities, and crime events per county were negatively correlated with food addiction symptoms (all r = –0.16, *p* < 0.01). By contrast, food addiction symptoms were positively correlated with multiple anthropometric outcomes, including BMI (r = 0.34, *p* < 0.01), waist circumference (r = 0.36, *p* < 0.01), hip circumference (r = 0.28, *p* < 0.01), and waist-to-hip ratio (r = 0.26, *p* < 0.01). We did not observe statistically significant correlations between environmental factors and CVD-related biomarkers in unadjusted analyses, except for triglycerides, which showed a correlation with the Food Environment Index (r = 0.12, *p* < 0.05). Additionally, waist-to-hip ratio was negatively correlated with number of crime events (r = −0.12, *p* < 0.05).

### 3.5. Results for Associations Between Environmental Factors and Food Addiction Symptoms

Regression models (Table 5) showed consistent associations between environmental factors and food addiction symptoms. A less favorable food environment, reflected by lower Food Environment Index scores, was significantly associated with higher food addiction symptoms in both unadjusted (β = –0.239, 95% CI: –0.323, –0.155) and BMI- and sex-adjusted models (β = –0.189, 95% CI: –0.205, –0.173). Greater counts of fast-food restaurants were also associated with slightly lower food addiction symptom scores in both unadjusted and adjusted models (β ≈ –0.000; 95% CIs ranged from –0.001 to 0.000), although the magnitude of these effects was minimal. Similarly, higher numbers of recreation and fitness facilities were inversely associated with food addiction symptoms in unadjusted (β = –0.003, 95% CI: –0.005, –0.001) and adjusted models (β = –0.002, 95% CI: –0.004, 0.000). Higher counts of crime events were likewise associated with marginally lower food addiction symptom scores, with small but statistically significant coefficients in both models. Sensitivity analyses using negative binomial regression to account for the count distribution of YFAS symptoms yielded results consistent in direction and inference with the primary analyses.

### 3.6. Results for Associations Between Food Addiction Symptoms and CVD-Related Biomarkers

Associations between food addiction symptoms and CVD-related biomarkers are presented in Table 6. In adjusted models, higher food addiction symptom counts were significantly associated with greater body weight (β = 0.012, 95% CI: 0.007–0.018), BMI (β = 0.085, 95% CI: 0.049–0.121), waist circumference (β = 0.046, 95% CI: 0.022–0.069), hip circumference (β = 0.044, 95% CI: 0.016–0.072), and waist-to-hip ratio (β = 6.732, 95% CI: 3.475–9.990). Food addiction symptoms were also associated with slightly lower triglyceride levels (β = –0.002, 95% CI: –0.003 to –0.000), although the magnitude of this association was small. No statistically significant associations were observed between food addiction symptoms and systolic blood pressure, diastolic blood pressure, mean arterial pressure, total cholesterol, HDL, LDL, or glucose concentrations in adjusted models.

### 3.7. Moderation Effects of Food Addiction Symptoms Between Environmental Factors and CVD-Related Biomarkers

Moderation analyses tested whether food addiction symptoms modified the relationship between environmental factors and CVD-related biomarkers (Supplementary Materials, Table S1). Most interaction terms were nonsignificant, indicating limited evidence of moderation. However, a significant interaction was observed between the Food Environment Index and food addiction symptoms in predicting waist-to-hip ratio (interaction β = 0.009, *p* = 0.03), although the effect sizes were small. This suggests that the association between Food Environment Index and waist-to-hip ratio may vary depending on the severity of food addiction symptoms. Additional marginal interactions were detected between the count of crime events and food addiction symptoms in predicting LDL cholesterol level (interaction β = –0.0005, *p* = 0.02), though effect sizes were small.

## 4. Discussion

This study investigated the relationships among environmental factors, food addiction symptoms, and CVD-related biomarkers in NC police officers. To our knowledge, this is the first study to examine the potential role of food addiction in linking environmental exposures with cardiometabolic health among law enforcement personnel, a high-risk occupational group facing both unique stressors and disproportionate CVD burden [70]. Overall, the findings suggest mixed results of the associations between environmental factors and food addiction symptoms, whereas food addiction symptoms were associated with obesity-related measures among police officers. However, further research is needed to clarify how structural inequities in food and recreation access interact with individual vulnerabilities to shape long-term cardiometabolic risk. Importantly, the absence of statistically significant correlations should not be interpreted as evidence of no underlying relationship, as these analyses were based on unadjusted correlations using county-level measures that may be subject to measurement error and ecological bias.

We found consistent evidence that county type and regional context shaped cardiometabolic risk indicators, environmental factors, and food addiction symptoms among police officers. In particular, rural officers exhibited significantly greater waist-to-hip ratios, worse food environment, less recreation and fitness facilities, alongside higher food addiction symptoms, relative to their urban and suburban counterparts. Although some of these differences reached statistical significance, the corresponding effect sizes (η^2^ < 0.01) were small, suggesting that the magnitude of these differences may be modest in terms of clinical relevance. Higher food addiction symptoms among rural officers may reflect greater food insecurity, longer travel distances to grocery stores, and limited availability of diverse food options, all of which have been linked to maladaptive eating behaviors [71,72,73]. Rural environments may also foster greater reliance on calorie-dense, shelf-stable, and convenience foods due to limited retail access and unpredictable work schedules [74]. These findings mirror broader population-level disparities in rural health, where limited access to recreation facilities [75,76], fewer healthcare resources [77], and higher occupational demands contribute to greater cardiometabolic risk [78]. Despite lower count of crime events in NC rural areas compared to urban and suburban areas from the finding, rural police agencies are typically much smaller, cover larger areas, patrol with fewer officers on shift, and face longer backup times—driving higher workload per officer and chronic operational stress [49]; this in turn may exacerbate disordered eating behaviors [79]. At the regional level, differences were less pronounced overall, but officers in the Mountain Region reported higher food addiction symptoms compared to those in the Piedmont and Coastal Plain Regions, despite having the lowest density of fast-food restaurants. One potential explanation is the limited availability of healthy food options and higher rates of food insecurity in mountain communities. The NC Mountain Region is the most rural part of the state, consisting of 2 suburban counties and 21 rural counties. No urban counties are located in this region, largely due to its remote geography and limited accessibility to many communities. The findings from this study align with prior research showing that rural areas often experience reduced access to supermarkets and must travel greater distances to reach high-quality grocery stores [80], factors that have been linked to food insecurity and food addiction [81]. Taken together, the unique geographic and socioeconomic challenges of the Mountain Region may exacerbate maladaptive eating behaviors, even in the absence of frequent fast-food exposure.

The findings from this study suggest that environmental exposures were significantly associated with food addiction symptoms, though in somewhat counterintuitive directions. Specifically, less favorable food environments—as reflected by lower Food Environment Index scores—were associated with higher food addiction symptoms. This finding is consistent with prior research showing that disadvantaged food environments, including food swamps and communities facing food insecurity with limited access to healthy foods, promote maladaptive eating behaviors and increase obesity risk [82,83]. We also found that a higher density of recreation facilities was associated with lower food addiction symptoms, which is consistent with previous studies reporting that greater access to recreational facilities influences physical activity and obesity risk [84], and that recreational facilities have the potential to influence dietary quality [85].

We found that higher densities of fast-food restaurants, recreation facilities, and crime events were unexpectedly associated with lower food addiction symptoms. This contrasts with prior research showing that greater accessibility to fast-food restaurants is linked to higher fast-food intake or binge-eating tendencies [86,87], and that greater availability of fast-food restaurants has been positively associated with violent crime rates across U.S. counties [88]. Urban counties may simultaneously offer greater access to both unhealthy and healthy food options, potentially diluting the isolated effect of fast-food density on eating behaviors. Additionally, officers’ work schedules may influence actual exposure to food environments. For example, night-shift officers may have limited access to restaurants that are not open 24 h, thereby reducing the practical impact of local fast-food density. One possible explanation is that county-level aggregate measures may not fully capture individual officers’ lived exposure to unhealthy food choices. For example, the findings from this study show that urban counties have a higher number of fast-food restaurants and more crime events compared to suburban and rural counties. At the same time, urban counties also score higher on the Food Environment Index, indicating better access to healthy food options. This means that officers working in high-crime or urban areas may encounter both greater exposure to unhealthy foods and greater availability of healthier choices, which could balance or reduce the overall impact of fast-food density on disordered eating behaviors. A review noted that neighborhoods with higher availability of unhealthy food outlets often simultaneously have greater access to healthy options, which complicates straightforward interpretations of food outlet density and dietary outcomes [89]. Additionally, officers’ irregular shifts may limit their use of local restaurants and increase reliance on convenient-based or pre-packed foods [32,90]. For example, most fast-food restaurants are not open 24/7, so night-shift officers may have little to no access to these options, reducing the influence of the surrounding fast-food environment on their eating patterns. It is worth noting that we did not account for officers’ exposure time within specific food environments or county population density, which may influence the degree to which environmental factors translate into individual behavior. The findings from this study underscore the complexity of environmental influences on disordered eating behaviors in law enforcement profession and highlight the need for more fine-grained, individual-level assessments of environmental exposure (e.g., geocoded dietary purchasing patterns, worksite food availability).

Consistent with the hypotheses, food addiction symptoms were positively associated with anthropometric markers of CVD risk factors, including weight, BMI, waist circumference, hip circumference, and waist-to-hip ratio. These findings align with meta-analytic evidence that food addiction is associated with elevated risk for vascular stroke [91], insulin resistance [92], hypertension and type 2 diabetes [93]. Notably, the associations between food addiction and blood pressure, lipid profiles, and glucose were not statistically significant in our sample except for triglyceride levels. This pattern may partly reflect the relatively young mean age of participants (38 years), as cardiometabolic dysregulation often develops progressively and may not yet be clinically manifest in early adulthood. Food addiction may therefore represent an early behavioral or metabolic phenotype that precedes overt hypertension or glycemic impairment. Prior cross-sectional and cohort evidence indicate that food addiction exerts its strongest and most immediate effects on adiposity-related measures, whereas downstream cardiometabolic consequences often emerge later in the disease trajectory [93,94,95]. Importantly, the absence of statistical significance does not necessarily indicate a lack of correlation or clinical relevance, and definitive conclusions should not be drawn on this basis alone. Rather, these findings should be interpreted within the context of a broader, more comprehensive evaluation of cardiometabolic risk. We also lacked data on hypertension treatment, dietary sodium intake, caffeine or energy drink consumption, which may influence blood pressure and glycemic outcomes and could attenuate observed associations. Future research would benefit from longitudinal designs and expanded biomarker panels—such as insulin resistance indices (e.g., HOMA-IR) and inflammatory markers (e.g., CRP)—to better characterize the temporal and biological pathways linking food addiction to cardiometabolic risk. Longitudinal studies are needed to clarify whether food addiction predicts worsening blood pressure and lipid outcomes over time.

Contrary to expectations, moderation analyses revealed limited evidence of interaction effects, with the notable exception that the relationship between the Food Environment Index and waist-to-hip ratio was stronger among individuals exhibiting increased food addiction symptoms. One interpretation is that in resource-rich settings, individuals with higher food addiction symptoms may be more exposed to abundant food options, amplifying the translation of addictive-like eating into central obesity. In one study with young adult women, higher food addiction symptoms were independently associated with greater visceral fat, a central-obesity marker more sensitive than BMI, suggesting addictive-like eating translates specifically into abdominal adiposity [96]. In another experiment with children, a varied, highly available obesogenic food environment interacted with impulsivity/reward sensitivity to increase intake, consistent with the idea that individuals high in appetitive traits consume more when options are abundant [97]. While preliminary, this finding raises important questions about how environmental exposure intersects with individual vulnerability to disordered eating behaviors in shaping downstream health consequences.

To our knowledge, this is the first investigation to examine associations between environmental exposures, food addiction symptoms, and CVD risk factors in a law-enforcement cohort. Nonetheless, several limitations warrant consideration. First, the environmental exposure data regarding fast-food restaurants and recreation/fitness facilities were derived from the most recent statewide dataset available (2016), whereas clinical data were collected between 2023 and 2025. This temporal mismatch may introduce exposure misclassification, as the number and geographic distribution of fast-food restaurants and recreation/fitness facilities may have changed over time. Consequently, county-level environmental measures may not fully reflect participants’ true exposure during the study period, potentially attenuating observed associations. These variables were retained in the analysis because, to our knowledge, no updated statewide dataset with comparable methodology, standardized definitions, and comprehensive geographic coverage is currently available. Preliminary findings from this study demonstrate patterns in the distribution of fast-food restaurants and recreation/fitness facilities across different county types and regions in NC, providing useful insight into historical geographic disparities in the built environment across NC. Future studies should prioritize the use of contemporaneous environmental data or longitudinal exposure tracking to reduce temporal misclassification and strengthen causal inference. Second, food addiction was assessed using a validated psychometric measure, but we did not collect objective nutritional intake data (e.g., consumption of high-fat or high-sugar foods). As a result, our findings reflect psychological and behavioral symptomatology rather than direct quantification of dietary patterns. The absence of dietary intake data limits our ability to examine how environmental factors influence food addiction at the behavioral consumption level beyond self-reported symptom characteristics. Future studies should incorporate objective dietary assessment methods such as 24 h food interviews during workday to better capture real-time eating behaviors and strengthen the behavioral validity of food addiction measurement. Third, LDL cholesterol was estimated using the Friedewald equation, which assumes triglyceride levels < 400 mg/dL and fasting state. While all samples were obtained in the fasting state, six participants exceeded this threshold. These individuals were retained to preserve statistical power; however, LDL values in these cases may be less reliable, potentially introducing minor measurement imprecision. Future studies may benefit from incorporating alternative lipid markers that are less influenced by elevated triglycerides, such as non–HDL cholesterol or apolipoprotein B, which are considered more stable and comprehensive indicators of atherogenic risk. Fourth, county-level measures of crime events were used in raw counts without standardization for population, geographic area, population density, or relative shares (e.g., ratio of fast-food outlets to fresh-food stores). Our study was limited to reported crime events from local law enforcement agencies and included only a subset of police officers rather than all officers across North Carolina’s 100 counties. Consequently, these environmental measures may not fully capture relative exposure levels and should be interpreted cautiously. Nevertheless, these preliminary findings provide an initial exploration of potential environmental influences, and future research with larger, more representative samples could incorporate standardized or ratio-based measures to more accurately assess environmental impacts. Fifth, the cross-sectional design precludes establishing temporal or causal ordering of observed associations. Future longitudinal or experimental studies are needed to confirm these mechanisms. Sixth, food addiction symptoms were assessed via self-report, which is subject to recall and social-desirability bias and does not constitute a clinical diagnosis. Seven, because the analysis used secondary data and was exploratory in nature, we did not conduct a priori power calculation to determine the minimum sample size. Although our achieved sample was moderate, subsequent phases of the Carolina Blue Project aim to expand recruitment and incorporate additional demographic, behavioral, occupational, and cardiometabolic measures. Finally, our sample consisted predominantly of male officers from NC’s Piedmont Region, which may limit generalizability to female officers and other geographic regions. Future work should employ larger, prospectively designed cohorts and integrate objective dietary-intake measures to more rigorously elucidate causal pathways and enhance external validity.

## 5. Conclusions

In conclusion, this study provides preliminary evidence that environmental factors may be linked to food addiction symptoms, which in turn may potentially be associated with adverse anthropometric measures among police officers. Although food addiction symptoms showed limited moderation of the associations between environmental factors and CVD risk indicators, they consistently emerged as a key independent variable of obesity-related outcomes. Considering the preliminary nature of these findings, future work should deepen our understanding using approaches that capture officers’ real-world food environments with greater precision, such as tracking where food is purchased or assessing the nutritional landscape within worksites. Moreover, future studies should use longitudinal cohorts with contemporaneous environmental data, objective dietary assessments, and more refined exposure metrics (e.g., standardized or ratio-based indicators and alternative lipid markers) to better clarify temporal relationships, reduce misclassification, and strengthen causal inference and generalizability.

## Figures and Tables

**Table 1:** Descriptive Statistics of All Variables.

Variable		n	Mean (SD)	n (%)
Age		330	37.98 (9.06)	
Sex		329		
	Male			221 (67.17%)
	Female			108 (32.83%)
Race		330		
	Caucasian			261 (79.09%)
	African American			48 (14.54%)
	Mixed Race			5 (1.52%)
	Other *			16 (4.85%)
Ethnicity		330		
	Non-Hispanic			310 (93.94%)
	Hispanic			12 (3.64%)
	Prefer not to answer			8 (2.42%)
County		330		
	Urban county			90 (27.27%)
	Suburban county			143 (43.33%)
	Rural county			97 (29.40%)
Region		330		
	Mountain region			56 (16.97%)
	Piedmont region			210 (63.64%)
	Coastal region			64 (19.39%)
BMI (kg/m^2^)		330	30.53(6.38)	
Waist circumference (cm)		330	103.06 (16.32)	
Hip circumference (cm)		330	113.10 (12.00)	
Waist-to-hip ratio		330	0.91 (0.09)	
Systolic blood pressure (mmHg)		330	127.15 (16.95)	
Diastolic blood pressure (mmHg)		330	84.60 (11.81)	
Mean arterial pressure (mmHg)		330	98.74 (12.64)	
Total cholesterol (mg/dL)		324	176.68 (37.70)	
Triglycerides (mg/dL)		299	123.01 (80.25)	
HDL (mg/dL)		326	44.97 (14.73)	
LDL (mg/dL)		330	103.73 (38.74)	
Glucose (mg/dL)		330	92.40 (27.12)	
Food environment index		330	7.72 (0.83)	
Food addiction symptoms		330	0.75 (1.80)	

Note. n = number of participants; SD = standard deviation; * Other = Native Hawaiian/Pacific Islander, Asian, American Indian/Native Alaskan, prefer not to answer, and other races specified by participants (n < 5). BMI = body mass index; kg = kilogram; m = meter; cm = centimeter; mmHg = millimeters of mercury; mg/dL = milligrams per deciliter; HDL = high-density lipoprotein cholesterol; LDL = low-density lipoprotein cholesterol.

**Table 2:** Descriptive Statistics of All Variables by County Types.

Variables	Urban County		Suburban County		Rural County		F	*p*-Value	ŋ^2^	Post hoc Test
	Mean	SD	Mean	SD	Mean	SD				
BMI (kg/m^2^)	30.54	5.58	30.05	5.64	31.41	7.89	0.91	0.34	<0.01	
Body weight (kg)	91.60	21.82	93.50	21.10	97.58	24.37	1.80	0.17	0.01	
Waist circumference (cm)	101.00	14.96	102.16	15.06	106.06	18.78	4.67	0.03	0.01	Rural > Urban (*p* = 0.08) Rural > Suburban (*p* = 0.16) Suburban > Urban (*p* = 0.85)
Hip circumference (cm)	113.28	11.57	112.69	10.35	113.43	14.44	0.13	0.88	<0.01	
Waist-to-hip ratio	0.89	0.08	0.90	0.09	0.93	0.10	5.38	<0.01	0.03	Rural > Urban ** Rural > Suburban * Suburban > Urban (*p* = 0.47)
Systolic blood pressure (mmHg)	126.54	18.20	127.25	14.91	127.59	18.41	0.10	0.91	<0.01	
Diastolic blood pressure (mmHg)	84.20	11.75	85.03	11.24	84.11	12.70	0.19	0.83	<0.01	
Mean arterial pressure (mmHg)	98.32	12.90	99.11	11.79	98.60	13.75	0.02	0.88	<0.01	
Total cholesterol (mg/dL)	174.27	42.42	180.96	36.44	172.42	34.04	1.68	0.19	0.01	
Triglycerides (mg/dL)	124.14	104.69	127.31	76.02	118.84	61.66	0.33	0.72	<0.01	
HDL (mg/dL)	45.76	14.89	45.44	15.02	43.52	14.11	0.67	0.51	<0.01	
LDL (mg/dL)	107.29	34.28	113.05	36.67	107.38	32.95	1.02	0.36	<0.01	
Glucose (mg/dL)	88.71	25.25	93.01	29.45	94.43	24.95	0.92	0.40	<0.01	
Food environment index	7.97	0.92	7.80	0.85	7.36	0.52	15.89	<0.01	0.12	Rural < Urban ** Rural < Suburban ** Suburban < Urban **
Count of fast-food restaurants	555.30	308.62	158.69	70.12	43.03	17.40	247.30	<0.01	0.60	Rural < Urban ** Rural < Suburban ** Suburban < Urban **
Count of recreation & fitness facilities	94.52	60.74	22.62	7.24	4.64	3.77	212.80	<0.01	0.57	Rural < Urban ** Rural < Suburban ** Suburban < Urban **
Count of crime events	19,032.57	7766.82	4835.49	2791.77	1127.81	689.98	429.10	<0.01	0.72	Rural < Urban ** Rural < Suburban ** Suburban < Urban **
Food addiction symptoms	0.47	1.22	0.61	1.65	1.21	2.31	4.64	0.01	0.03	Rural > Urban ** Rural > Suburban ** Suburban > Urban (*p* = 0.86)

Note. SD = standard deviation; F = F-statistic; ŋ^2^ = Partial eta-squared; BMI = body mass index; kg = kilogram; m = meter; cm = centimeter; mmHg = millimeters of mercury; mg/dL = milligrams per deciliter; HDL = high-density lipoprotein cholesterol; LDL = low-density lipoprotein cholesterol; * *p* < 0.05; ** *p* < 0.01.

**Table 3:** Descriptive Statistics of All Variables by Region Types.

Variables	Mountain Region		Piedmont Region		Coastal Region		F	*p*-Value	ŋ^2^	Post Hoc Test
	Mean	SD	Mean	SD	Mean	SD				
BMI (kg/m^2^)	29.93	8.59	31.07	5.96	29.61	5.27	0.13	0.72	<0.01	
Body weight (kg)	100.83	17.13	103.75	16.79	102.70	14.06	0.31	0.82	<0.01	
Waist circumference (cm)	100.83	17.13	103.60	16.73	102.70	14.06	0.33	0.56	<0.01	
Hip circumference (cm)	111.35	13.99	113.67	11.98	112.60	9.99	0.25	0.62	<0.01	
Waist-to-hip ratio	0.90	0.08	0.91	0.10	0.91	0.08	0.19	0.66	<0.01	
Systolic blood pressure (mmHg)	126.46	19.37	127.28	16.23	127.47	17.01	0.09	0.72	<0.01	
Diastolic blood pressure (mmHg)	83.88	12.93	84.90	11.09	84.05	13.17	<0.01	0.98	<0.01	
Mean arterial pressure (mmHg)	98.07	14.30	99.03	11.91	98.52	13.78	0.03	0.87	<0.01	
Total cholesterol (mg/dL)	169.83	33.74	176.98	38.04	182.17	38.59	2.98	0.09	<0.01	
Triglycerides (mg/dL)	107.77	48.93	130.15	90.33	116.51	69.52	0.27	0.60	<0.01	
HDL (mg/dL)	43.91	13.09	45.29	15.36	45.05	14.09	0.14	0.71	<0.01	
LDL (mg/dL)	104.84	36.04	109.35	33.48	116.73	37.50	1.51	0.22	<0.01	
Glucose (mg/dL)	93.02	20.36	91.93	24.99	92.59	37.51	<0.01	0.94	<0.01	
Food Environment Index	7.32	0.51	8.03	0.84	7.05	0.21	5.44	0.02	0.02	Coastal < Mountain ** Mountain < Piedmont ** Coastal < Piedmont **
Count of fast-food restaurants	47.86	12.84	292.32	310.03	210.05	74.02	14.26	<0.01	0.12	Coastal > Mountain ** Mountain < Piedmont ** Coastal < Piedmont **
Count of recreation & fitness facilities	5.32	2.74	51.29	55.90	19.50	7.97	19.69	<0.01	0.15	Coastal > Mountain ** Mountain < Piedmont ** Coastal < Piedmont **
Count of crime events	1192.04	413.20	9759.28	9694.19	6601.11	3365.70	17.7	<0.01	0.14	Coastal > Mountain ** Mountain < Piedmont ** Coastal < Piedmont **
Food addiction symptoms	1.45	2.51	0.61	1.54	0.58	1.71	3.50	0.02	0.03	Coastal < Mountain ** Mountain > Piedmont (*p* = 0.05) Coastal > Piedmont (*p* = 0.96)

Note. SD = standard deviation; F = F-statistic; ŋ^2^ = Partial eta-squared; BMI = body mass index; kg = kilogram; m = meter; cm = centimeter; mmHg = millimeters of mercury; mg/dL = milligrams per deciliter; HDL = high-density lipoprotein cholesterol; LDL = low-density lipoprotein cholesterol; ** *p* < 0.01.

**Table 4:** Correlation between Environmental Factors, Food Addiction Symptoms, and CVD-related biomarkers.

Variables	Food Environment Index	Count of Fast Food Restaurants	Count of Recreation & Fitness Facilities	Count of Crime Events	Food Addiction Symptoms
	r	r	r	r	r
BMI (kg/m^2^)	−0.01	−0.05	−0.04	−0.05	0.34 **
Waist circumference (cm)	0.02	−0.05	−0.05	−0.08	0.36 **
Hip circumference (cm)	0.05	0.02	0.02	−0.01	0.28 **
Waist-to-hip ratio	−0.01	−0.09	−0.08	−0.12 *	0.26 **
Systolic blood pressure (mmHg)	0.07	0.09	0.08	0.06	0.01
Diastolic blood pressure (mmHg)	0.03	0.04	0.04	0.04	−0.01
Mean arterial pressure (mmHg)	0.02	0.06	0.06	0.06	−0.03
Total cholesterol (mg/dL)	0.09	0.09	0.08	0.06	−0.07
Triglycerides (mg/dL)	0.12 *	−0.01	−0.001	−0.04	−0.07
HDL (mg/dL)	<−0.01	0.02	0.02	0.03	−0.08
LDL (mg/dL)	0.04	0.09	0.08	0.08	−0.05
Glucose (mg/dL)	0.04	−0.01	−0.01	−0.02	0.03
Food addiction symptoms	−0.12 *	−0.16 **	−0.16 **	−0.16 **	NA

Note. BMI = body mass index; kg = kilogram; m = meter; cm = centimeter; mmHg = millimeters of mercury; mg/dL = milligrams per deciliter; HDL = high-density lipoprotein cholesterol; LDL = low-density lipoprotein cholesterol; * *p* < 0.05; ** *p* < 0.01.

**Table 5:** Associations between Environmental Factors and Food Addiction Symptoms.

Independent Variables	Unadjusted			Adjusted		
	β	SE	95% CI	β	SE	95% CI
Food Environment Index	−0.239 *	0.043	(−0.323, −0.155)	−0.189 **	0.008	(−0.205, −0.173)
Count of fast-food restaurants	−0.000 **	0.000	(−0.001, −0.000)	−0.000 *	0.000	(−0.001, −0.000)
Count of recreation & fitness facilities	−0.003 *	0.001	(−0.005, −0.001)	−0.002 **	0.001	(−0.004, −0.000)
Count of crime events	−0.000 **	0.000	(−0.000, −0.000)	−0.000 **	0.000	(−0.000, −0.000)

Note. β = standardized parameter estimates; SE = standard error; 95% CI = 95% confidence interval; adjusted = model adjusted for BMI and sex; * *p* < 0.05; ** *p* < 0.01.

**Table 6:** Associations between Food Addiction Symptoms and CVD-Related Biomarkers.

Independent Variables	Unadjusted			Adjusted		
	β	SE	95% CI	β	SE	95% CI
Weight (kg)	0.009 **	0.002	(0.005, 0.014)	0.012 **	0.003	(0.007, 0.018)
BMI (kg/m^2^)	0.083 **	0.018	(0.047, 0.119)	0.085 **	0.018	(0.049, 0.121)
Waist circumference (cm)	0.039 **	0.011	(0.018, 0.061)	0.046 **	0.012	(0.022, 0.069)
Hip circumference (cm)	0.043 **	0.014	(0.015, 0.070)	0.044 **	0.014	(0.016, 0.072)
Waist to hip ratio	5.177 **	1.503	(2.220, 8.135)	6.732 **	1.656	(3.475, 9.990)
Systolic blood pressure (mmHg)	0.001	0.005	(−0.009, 0.012)	0.005	0.006	(−0.007, 0.018)
Diastolic blood pressure (mmHg)	−0.002	0.008	(−0.017, 0.014)	0.001	0.008	(−0.015, 0.018)
Mean arterial pressure (mmHg)	−0.000	0.007	(−0.013, 0.013)	0.004	0.008	(−0.011, 0.019)
Total cholesterol (mg/dL)	−0.003	0.003	(−0.008, 0.002)	−0.003	0.003	(−0.008, 0.002)
Triglycerides (mg/dL)	−0.002	0.000	(−0.003, −0.000)	−0.002 *	0.000	(−0.003, −0.000)
HDL (mg/dL)	−0.009	0.007	(−0.022, 0.005)	−0.016	0.009	(−0.034, 0.002)
LDL (mg/dL)	−0.003	0.003	(−0.008, 0.003)	−0.002	0.003	(−0.008, 0.004)
Glucose (mg/dL)	0.002	0.004	(−0.005, 0.009)	0.003	0.004	(−0.005, 0.011)

Note. β = standardized parameter estimates; SE = standard error; 95% CI = 95% confidence interval; adjusted = model adjusted for sex; BMI = body mass index; kg = kilogram; m = meter; cm = centimeter; mmHg = millimeters of mercury; mg/dL = milligrams per deciliter; HDL = high-density lipoprotein cholesterol; LDL = low-density lipoprotein cholesterol; * *p* < 0.05; ** *p* < 0.01.

## Data Availability

The research materials supporting this publication can be accessed by contacting Ya-Ke Wu.

## Supplementary Materials

The following supporting information can be downloaded at: https://www.mdpi.com/article/10.3390/ijerph23030311/s1, Table S1: Moderation Effects of Food Addiction Symptoms between Environmental Factors and CVD-Related Biomarkers.
