# Supplementary material for: Environmental Influences on Food Addiction and Cardiometabolic Profiles in Law Enforcement Officers
Source: Int J Environ Res Public Health. 2026 Mar 1;23(3):311. doi: 10.3390/ijerph23030311 (PMC13026730; doi:10.3390/ijerph23030311)
Supplement: Supplementary file 1 [file ijerph-23-00311-s001.zip › ijerph-4115248-supplementary.pdf]

Supplemental Table S1. Moderation Effects of Food Addiction Symptoms between Environmental Factors and CVD-Related Biomarkers.

Independent Variable	Moderator	Dependent variable	Interaction β	Interaction *p*-value	Adjusted R-squared
Food Environment Index	Food addiction symptoms	BMI (kg/m²)	3.22E-02	0.91	0.11
Food Environment Index	Food addiction symptoms	Weight (kg)	9.93E-01	0.65	0.09
Food Environment Index	Food addiction symptoms	Waist circumference (cm)	5.48E-01	0.46	0.13
Food Environment Index	Food addiction symptoms	Hip circumference (cm)	-5.73E-01	0.31	0.08
Food Environment Index	Food addiction symptoms	Wasit-to-hip ratio	9.39E-03	0.03	0.07
Food Environment Index	Food addiction symptoms	Systolic blood pressure (mmHg)	-5.76E-01	0.49	0.00
Food Environment Index	Food addiction symptoms	Diastolic blood pressure (mmHg)	-1.92E-01	0.49	0.00
Food Environment Index	Food addiction symptoms	Total cholesterol (mg/dL)	8.86E-01	0.64	0.00
Food Environment Index	Food addiction symptoms	Triglycerides (mg/dL)	3.79E+00	0.36	0.01
Food Environment Index	Food addiction symptoms	HDL (mg/dL)	2.45E-01	0.73	0.00
Food Environment Index	Food addiction symptoms	LDL (mg/dL)	-4.71E-01	0.79	-0.01
Food Environment Index	Food addiction symptoms	Glucose (mg/dL)	6.54E-01	0.62	-0.01
Count of fast-food restaurants	Food addiction symptoms	BMI (kg/m²)	-8.61E-04	0.47	0.11
Count of fast-food restaurants	Food addiction symptoms	weight (kg)	4.15E-03	0.65	0.09
Count of fast-food restaurants	Food addiction symptoms	Waist circumference (cm)	-4.26E-04	0.89	0.12
Count of fast-food restaurants	Food addiction symptoms	Hip circumference (cm)	1.05E-03	0.65	0.08
Count of fast-food restaurants	Food addiction symptoms	Wasit-to-hip ratio	-1.24E-05	0.48	0.06
Count of fast-food restaurants	Food addiction symptoms	Systolic blood pressure (mmHg)	-1.16E-03	0.73	0.00
Count of fast-food restaurants	Food addiction symptoms	Diastolic blood pressure (mmHg)	-3.88E-04	0.73	0.00

Count of fast-food restaurants	Food addiction symptoms	Total cholesterol (mg/dL)	-8.53E-03	0.26	0.01
Count of fast-food restaurants	Food addiction symptoms	Triglycerides (mg/dL)	3.77E-03	0.84	0.00
Count of fast-food restaurants	Food addiction symptoms	HDL (mg/dL)	2.26E-03	0.44	0.00
Count of fast-food restaurants	Food addiction symptoms	LDL (mg/dL)	-1.41E-02	0.08	0.01
Count of fast-food restaurants	Food addiction symptoms	Glucose (mg/dL)	-1.94E-03	0.72	-0.01
Count of crime events	Food addiction symptoms	BMI (kg/m^2)	-3.19E-05	0.37	0.11
Count of crime events	Food addiction symptoms	weight (kg)	4.26E-05	0.87	0.08
Count of crime events	Food addiction symptoms	Waist circumference (cm)	-3.53E-05	0.70	0.12
Count of crime events	Food addiction symptoms	Hip circumference (cm)	2.07E-05	0.76	0.07
Count of crime events	Food addiction symptoms	Wasit-to-hip ratio	-4.66E-07	0.37	0.07
Count of crime events	Food addiction symptoms	Systolic blood pressure (mmHg)	-3.25E-05	0.74	-0.01
Count of crime events	Food addiction symptoms	Diastolic blood pressure (mmHg)	-1.08E-05	0.74	-0.01
Count of crime events	Food addiction symptoms	Total cholesterol (mg/dL)	-3.65E-04	0.10	0.01
Count of crime events	Food addiction symptoms	Triglycerides (mg/dL)	-1.83E-05	0.97	0.00
Count of crime events	Food addiction symptoms	HDL (mg/dL)	6.93E-05	0.42	0.00
Count of crime events	Food addiction symptoms	LDL (mg/dL)	-5.15E-04	0.02	0.02
Count of crime events	Food addiction symptoms	Glucose (mg/dL)	-9.64E-05	0.55	-0.01
Count of recreation & fitness facilities	Food addiction symptoms	BMI (kg/m^2)	-2.30E-03	0.74	0.11
Count of recreation & fitness facilities	Food addiction symptoms	weight (kg)	4.28E-02	0.41	0.09
Count of recreation & fitness facilities	Food addiction symptoms	Waist circumference (cm)	-3.74E-04	0.98	0.12
Count of recreation & fitness facilities	Food addiction symptoms	Hip circumference (cm)	7.16E-03	0.59	0.08

Count of recreation & fitness facilities	Food addiction symptoms	Wasit-to-hip ratio	-6.32E-05	0.53	0.06
Count of recreation & fitness facilities	Food addiction symptoms	Systolic blood pressure (mmHg)	-3.45E-03	0.86	0.00
Count of recreation & fitness facilities	Food addiction symptoms	Diastolic blood pressure (mmHg)	-1.15E-03	0.86	0.00
Count of recreation & fitness facilities	Food addiction symptoms	Total cholesterol (mg/dL)	-3.89E-02	0.38	0.00
Count of recreation & fitness facilities	Food addiction symptoms	Triglycerides (mg/dL)	4.55E-02	0.67	0.00
Count of recreation & fitness facilities	Food addiction symptoms	HDL (mg/dL)	1.31E-02	0.44	0.00
Count of recreation & fitness facilities	Food addiction symptoms	LDL (mg/dL)	-7.57E-02	0.11	0.01
Count of recreation & fitness facilities	Food addiction symptoms	Glucose (mg/dL)	-5.81E-03	0.85	-0.01

Note. Interaction β = standardized parameter estimates for the interaction term between an independent variable and a moderator; Interaction *p*-value = the *p*-value for the interaction term between an independent variable and a moderator. HDL = high-density lipoprotein cholesterol; LDL = low-density lipoprotein cholesterol; adjusted = model adjusted for BMI and sex.
